# O7.2. BREAKTHROUGH ON ANTIPSYCHOTIC MAINTENANCE MEDICATION IN A CLINICAL COHORT

**DOI:** 10.1093/schbul/sby015.231

**Published:** 2018-04-01

**Authors:** Jose Rubio, Christoph Correll, Anil Malhotra, Majnu John, John Kane

**Affiliations:** 1 Northwell Health; 2 Zucker Hillside Hospital

## Abstract

**Background:**

Antipsychotic drugs are effective in reducing the severity of psychotic symptoms both in the short and long term, and in reducing risk of relapse. However, some patients may develop a relapse of their psychotic symptoms despite continued antipsychotic treatment. Arguably, this phenomenon would be best studied in patients treated with long-acting injectable (LAI) formulations, where the dates of exposure can be confirmed, removing the potential confounder of non-adherence. The characterization of this phenomenon can add important knowledge about the intrinsic efficacy of antipsychotic drugs, potential mechanisms involved in the decrement of their efficacy, and the underlying pathophysiology of psychosis that is not modulated via primarily dopaminergic mechanisms. Despite the implications of this clinical phenomenon, research on breakthrough on antipsychotic maintenance medication (BAMM) in models not confounded by non-adherence has been limited. To date, little is known about the incidence and predictors of BAMM in clinical populations.

**Methods:**

We extracted data from a cohort of individuals with a psychotic disorder who were initiated on their first LAI treatment between 2010 and 2015 in the injection clinic at The Zucker Hillside Hospital (New York, USA). We defined BAMM as hospitalization during the period of continuous treatment with LAI, which we used as the primary outcome. LAI treatment was considered continuous for each treatment episode if it was administered following the manufacturer’s recommendations for the first 2 months, and until there was a delay in the administration that would have required additional oral supplementation according to the manufacturer instructions (typically >1.5 times the scheduled interval of administration). We measured the cumulative incidence and time to BAMM in individuals with continuous LAI administration, and conducted univariate and multivariate analyses of covariates.

**Results:**

A total of 291 episodes of continuous treatment were observed. Of those, 44 (15.1%) led to hospitalization despite continuous treatment with a LAI antipsychotic. The median time to hospitalization was 204.5 days. In the multivariate analysis, the number of hospitalizations prior to onset of LAI treatment (5 vs 2, OR=2.75; 95%CI=1.60–4.72) and time since last hospitalization (4 vs 24.8 weeks, OR 0.70; 95%CI=0.53–0.91) were significantly associated with greater odds of hospitalization during continuous antipsychotic treatment. Individuals who were hospitalized despite continuous treatment were more likely to subsequently be treated with clozapine or ECT (18.2% vs 0, OR=4.93; 95%CI=1.25–19.40). We conducted a mutivariate Cox regression analysis for time to hospitalization and a sensitivity analysis comparing BAMM with individuals that completed 2 years of continuous treatment without being hospitalized and the results were consistent.

**Discussion:**

In a clinical cohort, a meaningful proportion of patients with a psychotic disorder treated with LAIs were hospitalized, despite confirmed continuous treatment. The median time to this event occurred about 7 months after onset of LAI treatment, suggesting that these patients had been stable and had reached steady state antipsychotic levels prior to hospitalization. Patients with a more active illness at the time of initiation of LAI treatment were more likely to relapse. These data suggest that more comprehensive investigation of BAMM is feasible; and therefore research focused on this unique group of individuals may provide novel insights into the pathophysiology of psychosis and into the mechanism of action of antipsychotic drugs.

